# The ubiquitin hydrolase, USP22 contributes to 3’-end processing of JAK-STAT inducible genes

**DOI:** 10.1186/1756-8935-6-S1-P27

**Published:** 2013-03-18

**Authors:** Edmond Chipumuro, Melissa A Henriksen

**Affiliations:** 1 Department of Biology, University of Virginia, Charlottesville, VA, USA

## 

5’-capping, splicing and 3’-cleavage/polyadenylation of a nascent RNA transcript are all coupled to transcription. Transcription is regulated by the post-translational modification of histones at the chromatin template. How histone modifications affect cotranscriptional RNA processing, however, is not well understood. By studying how the chromatin template contributes to the dynamic gene expression induced downstream of the JAK-STAT signaling pathway, we have uncovered relationships between specific histone modifications and RNA processing. Here we report that H2B monoubiquitination (ubH2B) is required for the effective 3’-end processing of the JAK-STAT inducible transcript, *IRF1*. RNAi-mediated depletion of the ubiquitin hydrolase, USP22 increases ubH2B levels and decreases transcriptional elongation at *IRF1*. Unexpectedly, 3’-end cleavage and polyadenylation of *IRF1* is diminished, leading to a 2-to-3-fold increase in unprocessed *IRF1* transcripts. The polyadenylation factor, CPSF73 is not effectively recruited and serine 2 phosphorylation (Ser2P) of the C-terminal domain of RNA polymerase II is disrupted. Two other JAK-STAT inducible transcripts are similarly affected, while two constitutively expressed transcripts are not. A working model, wherein a cycle of H2B ubiquitination/deubiquitination specifies Ser2P to regulate elongation and 3’-end processing of JAK-STAT inducible mRNAs is proposed. These results further elaborate USP22 function and its role as a putative cancer stem cell marker.

